# Retraction Note: Psychometric properties of the self-report version of the strengths and difficulties questionnaire in the Ecuadorian context: an evaluation of four models

**DOI:** 10.1186/s40359-020-00487-1

**Published:** 2020-12-18

**Authors:** Paúl Arias-Medina

**Affiliations:** Faculty of Psychology, University of Cuenca, Cuenca, Ecuador

## Correction to: *BMC Psychol* (2019) 7:51 10.1186/s40359-019-0328-6

The authors have retracted this article [1] because the Strengths and Difficulties Questionnaire has been modified without permission from the copyright holder youthinmind. The content of the article is no longer available owing to this breach.

All authors agree with this retraction.
